# A Practical Manual, Containing a Description of the General, Chemical, and Microscopical Characters of the Blood and Secretions of the Human Body, as Well as of Their Components, Including Both Their Healthy and Diseased States, with the Best Methods of Separating and Estimating Their Ingredients; Also, a Succinct Account of the Various Concretions Occasionally Found in the Body, and Forming Calculi

**Published:** 1847-01

**Authors:** 


Art. XXII.—*A Practical Manual, containing a Description of the General, Chemical. and Microscopical Characters of the Blood and Secretions of the Human Body, as well as of their Components, including both their Healthy and Diseased States, with the best methods of separating and estimating their Ingredients; also, a succinct Account of the various Concretions occasionally found in the Body, and forming Calculi.* By John W. Griffith, m.d., f.l.s., Senior Physician to the Finsbury Dispensary.—*London,* 1846. Part II. 12mo, pp. 168. With two Copper-plates.

We noticed the former part of Dr. Griffith's Manual soon after its publication, three years since, and then commended it as valuable so far as it went, though we felt called upon to express our surprise that so ample a title-page should be the prelude to so partial a survey of the subjects announced. In the little volume now before us, however, we have the completion of the entire range; and this in a very satisfactory manner. We can only regret, on Dr. Griffith's account, that he did not publish the whole work at once; as it would then have possessed an equality of character which it now wants, the interval between the two parts having been one of much progress in regard to the subjects of the first, and would doubtless have commanded a much more extensive sale, the public having a very reasonable objection to a work issued in parts. We can strongly recommend it, in its now complete state, to the attention of our readers; not as superseding larger and more elaborate treatises, but as presenting, in a very compendious form, a great quantity of most valuable information. The following apposite remarks form part of the preface to the present part:

"The important light which has been thrown upon several points in physiology and pathology, by the researches of modern chemistry and microscopy, are so striking that to be alone acquainted with them is sufficient to ensure a due appreciation of their importance. To argue that such investigations are idle, merely because each new truth which is elicited is not immediately applicable to the elucidation of some point in the history of a disease, or to the improved application of remedial means for its alleviation, is as absurd as unfortunately it is frequent. There is, however, one consolation in this matter; which is, that those who are the most ready to urge these views, and to decry the utility of calling in the aid of the collateral sciences, are such as are least acquainted with their details." (p. v.)

